# Single-Stage Combined Embolization and Resection for Spetzler-Martin Grade III/IV/V Arteriovenous Malformations: A Single-Center Experience and Literature Review

**DOI:** 10.3389/fneur.2020.570198

**Published:** 2020-10-29

**Authors:** Yu Chen, Ruinan Li, Li Ma, Yang Zhao, Tengfei Yu, Hao Wang, Xun Ye, Rong Wang, Xiaolin Chen, Yuanli Zhao

**Affiliations:** ^1^Department of Neurosurgery, Beijing Tiantan Hospital, Capital Medical University, Beijing, China; ^2^China National Clinical Research Center for Neurological Diseases, Beijing, China; ^3^Stroke Center, Beijing Institute for Brain Disorders, Beijing, China; ^4^Beijing Key Laboratory of Translational Medicine for Cerebrovascular Disease, Beijing, China; ^5^Department of Neurosurgery, Peking University International Hospital, Peking University, Beijing, China; ^6^Beijing Translational Engineering Enter for 3D Printer in Clinical Neuroscience, Beijing, China

**Keywords:** arteriovenous malformation, hybrid angio-surgical suite, embolization, microsurgical resection, outcomes, embolization degree

## Abstract

**Background and Purpose:** This study sought to identify the efficacy and intraoperative operational details of single-stage combined embolization and microsurgery strategy for Spetzler-Martin (SM) grade III/IV/V arteriovenous malformations (AVMs).

**Methods:** The authors retrospectively reviewed consecutive SM grade III/IV/V AVMs who underwent hybrid procedures and surgical resection alone procedures from January 2016 to February 2018. Outcomes [modified Rankin Scale (mRS)] were compared between hybrid group and surgical resection alone group in ruptured or unruptured subgroup. Factors associated with long-term disability were assessed using multivariable logistic regression analyses.

**Results:** A total of 100 AVM patients (47 corrected using hybrid procedures whereas 53 by surgical resection alone) were evaluated. After a mean follow-up of 2.3 ± 0.6 years, we found no difference in long-term prognosis and incidences of disability rates between these two strategies. However, the hybrid strategy offers significant advantage in accelerating the resection process [ruptured (*P* = 0.000); unruptured (*P* = 0.002)]. In the analysis of risk factors, excessive embolization (Grade C, 60–100%) was significantly associated with long-term disability in the hybrid cohorts (*P* = 0.041; odds ratio, 24.000; 95% CI, 1.140–505.194), and involvement of deep perforating arteries was the significant predictor of long-term disability in the surgical resection alone cohort (*P* = 0.025; odds ratio, 15.389; 95% CI, 1.412–167.66). In the subgroup analysis of the hybrid cohort, moderate embolization (Grade B, 30–60%) was recommended because of the low risk ratio of major intraoperative bleeding (*P* = 0.033).

**Conclusions:** Single-stage combined embolization and resection is an efficient strategy for the treatment of SM grade III/IV/V AVMs. Although the long-term outcomes were similar to surgical resection alone, the hybrid strategy had obvious advantages of shorter resection. In the hybrid technique, moderate embolization was recommended, and excessive embolization might be detrimental to the subsequent microsurgical resection.

**Clinical Trial Registration:** http://www.clinicaltrials.gov. Unique identifier: NCT04136860.

## Introduction

Arteriovenous malformations (AVMs) are complex and rare cerebral vascular dysplasia. Treatment interventions mainly aims at preventing the neurological impairment resulting from hemorrhagic stroke (1). The Spetzler-Martin (SM) grading system is widely used to estimate the risk of postoperative complication (2). Generally, SM grade I/II are amenable to surgical resection alone. Endovascular embolization and radiosurgery (SRS) have also been shown to achieve high recoveries with limited morbidity and mortality for low-level AVMs. Grade III AVMs typically require multimodal approach, including microsurgical resection, embolization, and SRS. Grade IV and V are generally monitored unless ruptured (3). However, previous studies have shown that despite the high rate of poor outcomes for high-level unruptured AVMs, the associated mortalities are likely lower than untreated patients (4, 5). Recently, several studies proposed that the intraoperative embolization strategies might benefit the subsequent surgical resection procedure and the long-term neurofunctional outcomes for complex cerebral AVMs (6–14). In this study, we assessed the prognostic difference between hybrid and surgical resection alone strategy in obliterating SM grade III–V AVMs, and we further explored the intraoperative embolization degree and resection portion surrounding the single-stage hybrid strategy.

## Materials and Methods

### Study Design and Participants

All brain AVM patients who underwent resection procedure (including hybrid strategy and surgical resection alone strategy) in Peking University International Hospital between January 2016 and February 2018 were retrieved from our institutional database. The study was performed according to the guideline of the Helsinki Declaration and was approved by the ethics committee of Peking University International Hospital. The inclusion criteria were as follows: (1) Diagnosed with AVM by digital subtraction angiography (DSA) and/or magnetic resonance imaging (MRI); (2) Patients received surgical resection alone or single-stage combined embolization and resection; (3) Patients with SM grade III–V; (4) Followed-up for more than 1 year. Patients with multiple AVMs, hereditary hemorrhagic telangiectasia (HHT), or who underwent invasive procedures (microsurgery, embolization, radiosurgery) before admission were excluded from the study.

### Data Collection and Definition

Demographic characteristics, clinical features, and imaging data were collected as baseline. Surgical resection alone was defined as resection without embolization. The definition of eloquent area and deep venous drainage were consistent with the Spetzler-Martin (SM) Grading system (2). Diffuseness was determined by preoperative angiograms with TOF images used to identify intervening brain parenchyma within the nidus. The degree of embolization was assessed by two professional neuroradiological physicians based on a three-stage grading system according to the changes of lesion volume and blood flow: Grade A, 0–30%; Grade B, 30–60%; Grade C, 60–100%. Disagreements in the embolization degree were arbitrated by a third physician. The intraoperative blood loss and operational duration were also collected. The perioperative complications such as postoperative hemorrhage, intracranial infection, and new-onset neurological dysfunction were evaluated within one week after the operation. The neurofunctional prognosis was analyzed based on modified Rankin Scale (mRS). The long-term outcomes were evaluated at the last follow-up. MRS > 2 was considered as postoperative neurological disability.

Follow-up was conducted within the first 3–6 months and annually after discharge through clinical visits and telephone interviews. Angiography was performed 1 year after the operation. The mRS scoring was conducted by neurosurgeons with at least 5 years' experience of clinical practice. All radiographic images were interpreted independently by at least two radiologists with equally at least 5 years of clinical experiences in the radiology center of our institute. Researchers who performed follow-up assessments were blinded to treatment modalities.

### Hybrid Procedure

Neurosurgeons and interventional neuroradiologists prospectively planned the surgical procedure of each AVM at the multidisciplinary vascular conference held every weekday in our center. Large nidus (>3 cm), deep location, multi-source blood supply, and involved critical eloquent areas were considered as complex AVMs. After a comprehensive analysis of the angioarchitectural and hemodynamic characteristics, some complex cases were recommended for hybrid strategy (single-stage combined embolization and microsurgical resection).

The operation was performed in a hybrid angio-surgical suite consisting of a surgical microscope (Pentero 900, Carl Zeiss Surgical AG, Oberkochen, Germany) and a flat biplane panel of digital subtraction angiography unit (AlluraXper20, Philips Healthcare, the Netherlands). All procedures were performed after induction of general anesthesia. Systolic blood pressure was maintained between 120 and 140 mmHg. A 6-Fr catheter was used to access to lesions and support the diagnostic angiography and embolization. The main polymeric embolic agent used on the study participant was Onyx 18 (eV3, Inc.), which contains 6% ethylene vinyl alcohol and 94% dimethyl sulfoxide. A bifemoral approach was preferred when the nidus was supplied by two different circulations (for example, contralateral supply or both anterior and posterior circulation), together with 2 guiding catheters (Envoy 6F, Cordis) placed accordingly.

During the embolization, we preferred to embolize the following parts: (1) the largest and straightest feeding arteries and its supplying unit, (2) part with high rupture risk (flow-related aneurysms or high-flow arteriovenous fistula), (3) expanded deep perforating arteries, (4) extracranial blood supply from the dura mater. When intra-embolization rupture happens, XperCT (AlluraXper20, Philips Healthcare, the Netherlands) of the angiogram machine would be performed to detect the hemorrhage and external ventricular drainage (EVD) placement might be performed according to the hemorrhage location and intracranial pressure, and then, the subsequent resection and hematoma evacuation would be done instantly.

After the embolization, the femoral artery sheath was retained, the guide catheter was removed, and the injection of heparin saline was stopped. The microsurgical procedures would then be performed with the assistance of intraoperative neuronavigation, ultrasonography, indocyanine fluorescence angiography (ICG), continuous monitoring of electroencephalogram, and somatosensory evoked potential. Circumferential dissection was performed along the borders of AVM within the perinidal gliotic tissue. Complete resection of embolized component and residual lesions were attempted when safe. If electrophysiological monitoring abnormalities occurred during the resection, the embolized parts adjacent to the eloquent area were preferably retained. Repeated electrocoagulation of the severed end of the embolized feeding arteries was performed to avoid postoperative recanalization. After the resection and before closing the skull, cerebral angiograms through the retained femoral artery sheath were performed to identify the completely occlusion of the lesion. If residues were detected, then secondary resection would be performed immediately.

A standardized perioperative management protocol was applied to all cases, with postoperative systolic blood pressure maintained between 120 and 140 mmHg, and antiepileptic and anti-cerebral edema treatments performed routinely after the operation. All patients underwent a computed tomography (CT) scan at postoperative day 1 to detect whether postoperative acute hemorrhage or infarction occurred. A MRI scan was performed at postoperative day 3. Repeat CT or MRI scans were performed if patients experienced severe headache or postoperative neurological deterioration.

### Statistical Analysis

The categorical variables were presented as counts (with percentages), whereas the continuous variables are presented as the means ± standard deviations (normal distribution variable) or median (quartile) (non-normal distribution variable). Two-tailed *t*-tests or one-way ANOVA were employed to compare normal distribution continuous variables, and Mann–Whitney *U*-test was applied to compare non-normal distribution continuous variables. The Pearson chi-square test or Fisher exact test were used to compare categorical variables as appropriate. A stratified analysis of pediatrics and adults was conducted to compare the intraoperative blood loss. Bonferroni correction was adopted in the adjusted *post-hoc* analysis in order to avoid the occurrence of Type I errors in the subgroup analysis of embolization degree (*P* < 0.005). Odds ratios (ORs) and 95% confidence intervals (CIs) for potential risk factors of postoperative disability in the hybrid group were calculated by univariate and multivariable logistic regression analyses. *P* < 0.05 was considered to be statistically significant. Statistical analyses were performed using SPSS (version 25.0, IBM).

## Results

### Baseline Characteristics

A total of 100 consecutive AVM patients (47 corrected using hybrid procedures whereas 53 by surgical resection alone) were included in this study. The mean age was 30.1 ± 13.0 years (range 4.6–61.7 years). Clinically, more than half of the patients (53.0%) were presented with hemorrhage (hybrid vs. surgical resection alone: 55.3 vs. 50.9%). Fifty-nine percent were evaluated as SM grade III (hybrid vs. surgical resection alone: 55.3 vs. 62.3%). In the hybrid group, 33 patients had a reduced SM grade after embolization (17 cases were SM grade II, 24 cases were SM grade III, and 6 cases were SM grade IV). The overall mean mRS scores at admission was 1.2 ± 0.5 (hybrid vs. surgical resection alone: 1.2 ± 0.4 vs. 1.2 ± 0.5). The average size of the nidus was 4.7 ± 1.5 cm (range 2.09–8.62 cm) (hybrid vs. surgical resection alone: 4.7 ± 1.3 vs. 4.8 ± 1.7 cm). Because of the priority of hybrid strategy for deep lesions, the hybrid group was more likely to be accompanied with deep venous drainage (hybrid vs. surgical resection alone: 57.4 vs. 37.7%, *P* = 0.049). The other angiographic characteristics between these two groups were similar (Table 1).

**Table 1:** Baseline demographic and angiographic characteristics.

**Characteristics**	**All patients (*n* = 100)**	**Hybrid (*n* = 47)**	**Surgical resection alone (*n* = 53)**	***P*-value**
Age (years)	30.1 ± 13.0	29.7 ± 11.7	31.6 ± 14.9	0.633
Sex (male)	59 (59.0%)	26 (55.3%)	33 (62.3%)	0.481
Manifestation symptom				0.455
Hemorrhage	53 (53.0%)	26 (55.3%)	27 (50.9%)	0.662
Seizure	29 (29.0%)	11 (23.4%)	18 (34.0%)	0.246
Others	18 (18.0%)	10 (21.3%)	8 (15.1%)	0.422
Hypertension	6 (6.0%)	2 (4.3%)	4 (7.5%)	0.787
Admission mRS	1.2 ± 0.5	1.2 ± 0.4	1.2 ± 0.5	0.957
Spetzler-Martin Grade				0.675
III	59 (59.0%)	26 (55.3%)	33 (62.3%)	
IV	36 (36.0%)	19 (40.4%)	17 (32.1%)	
V	5 (5.0%)	2 (4.3%)	3 (5.7%)	
Side (left)	53 (53.0%)	25 (53.2%)	28 (52.8%)	0.971
AVM size (cm)	4.7 ± 1.5	4.7 ± 1.3	4.8 ± 1.7	0.615
Eloquence	80 (80.0%)	35 (74.5%)	45 (84.9%)	0.193
Deep venous drainage	47 (47.0%)	27 (57.4%)	20 (37.7%)	0.049^*^
Aneurysms (flow-related)	29 (29.0%)	18 (38.3%)	11 (20.8%)	0.054
Diffuse nidus	29 (29.0%)	17 (36.2%)	12 (22.6%)	0.137
Deep perforating arteries	41 (41.0%)	22 (46.8%)	19 (35.8%)	0.266
Follow-up time (years)	2.3 ± 0.6	2.4 ± 0.6	2.2 ± 0.5	0.137

*AVM, Arteriovenous Malformation; mRS, modified Rankin Scale*.

* *Statistical significance (P <0.05)*.

### Postoperative Outcomes

**Figure 1:**
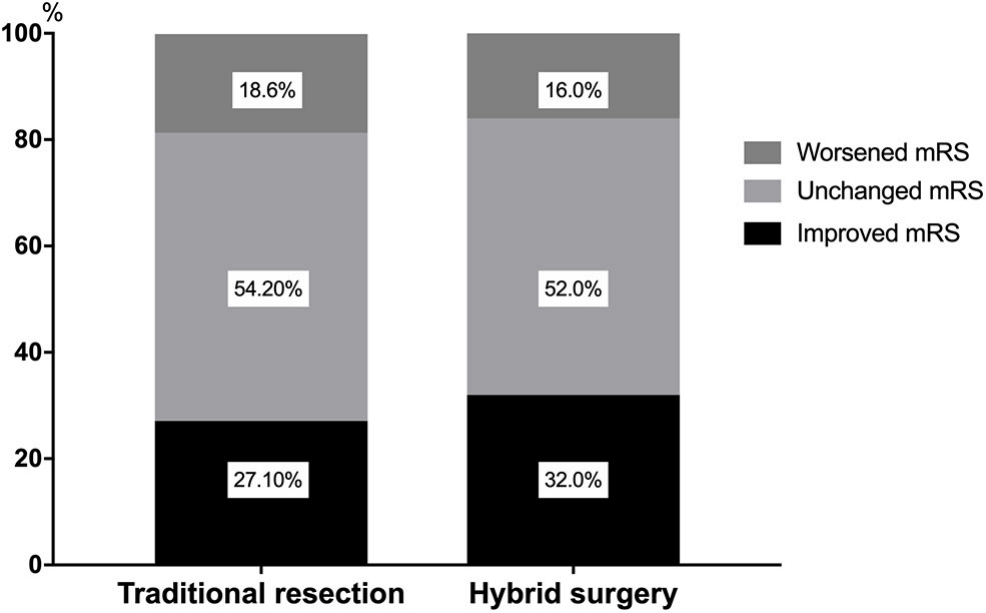
The changes of long-term outcomes in the surgical resection alone cohort and the single-stage hybrid cohort.

Continuous clinical or angiographic follow-up was performed for an average of 2.3 ± 0.6 years (range 1.2–3.3 years) for all the patients. In the ruptured cohorts, 26 patients (55.3%) underwent hybrid procedures, whereas 27 patients (50.9%) underwent surgical resection alone. Hybrid procedures (embolization+resection) lasted significantly longer than the surgical resection alone (7.3 ± 2.5 vs. 5.7 ± 2.0 h, *P* = 0.012; odds ratio, 95.918; 95% CI, 21.945–169.892). However, the actual surgical resection for the hybrid strategy was significantly shorter (3.7 ± 1.2 vs. 5.7 ± 2.0 h, *P* = 0.000; odds ratio, 119.735; 95% CI, 66.247–173.223). There were no significant differences in intraoperative blood loss (*P* = 0.059) and short-term outcomes (*P* = 0.453) between the two strategies. In the pediatric subgroup, the intraoperative blood loss was also similar between these two strategies (ruptured: *P* = 0.254; unruptured: 0.629). Intraoperative angiography revealed residual lesion in 1 patient (3.8%) in the hybrid group, which was successfully corrected by a subsequent resection before the skull closure of the first resection. During the perioperative period, 3 patients (11.5%) in the hybrid group presented with delayed postoperative hemorrhage. One of them received emergency hematoma evacuation surgery, and no recanalization was observed, and the other two patients received conservative medical treatment. In addition, one other patient in the surgical resection alone group experienced postoperative hemorrhage arising from residual lesion (found by postoperative DSA), and this patient subsequently received conservative treatment. Overall, after follow-up of on average 2.3 years, no significant differences were found regarding long-term mRS (0.9 ± 1.0 vs. 1.1 ± 1.1, *P* = 0.373) and long-term obliteration rates between these two groups (100.0 vs. 96.2%, *P* = 1.000) (Figure 1). Eight patients (15.1%) in the ruptured cohorts suffered long-term disabilities (mRS > 2) (hybrid surgery vs. surgical resection alone: 3 [11.5%] vs. 5 [18.5%], *P* = 0.745).

For the cohort with unruptured AVM, 21 cases (44.7%) received hybrid procedures whereas 26 patients (49.1%) underwent surgical resection alone. Although the total surgical duration for the two strategies was similar (*P* = 0.615), the actual resection duration for the hybrid group was significantly shorter than the surgical resection alone (3.5 ± 1.4 vs. 5.7 ± 2.7 h, *P* = 0.002; odds ratio, 132.447; 95% CI, 52.994–211.899). There was no significant difference between both short-term (1.5 ± 0.9 vs. 1.7 ± 1.2, *P* = 0.599) and long-term mRS (0.5 ± 1.0 vs. 0.8 ± 1.4, *P* = 0.313) and long-term disability (mRS > 2, 9.5 vs. 7.7%, *P* = 1.000) between these two groups. One patient in the surgical resection alone group succumbed to intraoperative hemorrhagic shock (intraoperative blood loss: 11,500 ml) and died (Table 2).

**Table 2:** Comparison of clinical outcomes between hybrid group and surgical resection alone group in ruptured AVMs and unruptured AVMs.

**Characteristics**	**Ruptured**		***P*-value**	**Unruptured**		***P*-value**
	**Hybrid (*n* = 26)**	**Surgical resection alone (*n* = 27)**		**Hybrid (*n* = 21)**	**Surgical resection alone (*n* = 26)**	
Sex (male)	17 (65.4%)	17 (63.0%)	0.854	9 (42.9%)	16 (61.5%)	0.202
Age (years)	31.0 ± 13.5	28.8 ± 13.1	0.560	28.1 ± 8.9	33.1 ± 14.4	0.172
Hypertension	2 (7.7%)	1 (3.7%)	0.973	0 (0.0%)	3 (11.5%)	0.313
Admission mRS	1.3 ± 0.5	1.3 ± 0.7	0.689	1.1 ± 0.4	1.1 ± 0.3	0.477
Side (left)	16 (61.5%)	12 (44.4%)	0.213	9 (42.9%)	16 (61.5%)	0.202
Size (cm)	4.5 ± 1.1	4.3 ± 1.4	0.475	4.9 ± 1.5	5.4 ± 1.7	0.253
Eloquence	21 (80.8%)	22 (81.5%)	1.000	14 (66.7%)	23 (88.5%)	0.145
Deep venous drainage	16 (61.5%)	13 (48.1%)	0.328	11 (52.4%)	7 (26.9%)	0.074
Spetzler-Martin Grade			0.177			0.926
III	14 (53.8%)	19 (70.4%)		12 (57.1%)	14 (53.8%)	
IV	12 (46.2%)	7 (25.9%)		7 (33.3%)	10 (38.5%)	
V	0 (0.0%)	1 (3.7%)		2 (9.5%)	2 (7.7%)	
Aneurysms (flow-related)	9 (34.6%)	5 (18.5%)	0.184	9 (42.9%)	6 (23.1%)	0.148
Diffuse nidus	12 (46.2%)	8 (29.6%)	0.215	5 (23.8%)	4 (15.4%)	0.721
Deep perforating arteries	16 (61.5%)	11 (40.7%)	0.130	6 (28.6%)	8 (30.8%)	0.870
Follow-up time (years)	2.3 ± 0.7	2.2 ± 0.5	0.483	2.4 ± 0.6	2.2 ± 0.5	0.204
Operation duration (hours)	7.3 ± 2.5	5.7 ± 2.0	0.012^*^	6.1 ± 1.5	5.7 ± 2.7	0.615
Resection duration (hours)	3.7 ± 1.2	5.7 ± 2.0	0.000^*^	3.5 ± 1.4	5.7 ± 2.7	0.002^*^
Intraoperative blood loss (ml)	550 (450)	750 (700)	0.059	500 (750)	575 (975)	0.259
Postoperative intracranial infection	7 (26.9%)	3 (11.1%)	0.263	5 (23.8%)	3 (11.5%)	0.470
Postoperative infarction	0 (0.0%)	0 (0.0%)	1.000	1 (4.8%)	0 (0.0%)	0.914
Postoperative hemorrhage	3 (11.5%)	1 (3.7%)	0.576	0 (0.0%)	0 (0.0%)	1.000
Perioperative subsequent surgery	1 (3.8%)	0 (0.0%)	1.000	0 (0.0%)	0 (0.0%)	1.000
New-onset visual disturbance	1 (3.8%)	0 (0.0%)	0.985	1 (4.8%)	3 (11.5%)	0.763
New-onset aphasia	1 (3.8%)	1 (3.7%)	1.000	1 (4.8%)	2 (7.7%)	1.000
New-onset Weakness	5 (19.2%)	9 (33.3%)	0.244	2 (9.5%)	4 (15.4%)	0.874
Short-term mRS	1.8 ± 1.0	2.1 ± 1.1	0.453	1.5 ± 0.9	1.7 ± 1.2	0.599
Long-term obliteration rate	100.0%	96.3%	1.000	100.0%	100.0%	1.000
Long-term mRS	0.9 ± 1.0	1.1 ± 1.1	0.373	0.5 ± 1.0	0.8 ± 1.4	0.313
Long-term disability (mRS > 2)	3 (11.5%)	5 (18.5%)	0.745	2 (9.5%)	2 (7.7%)	1.000
Death	0 (0.0%)	0 (0.0%)	1.000	0 (0.0%)	1 (3.8%)	1.000

*AVM, Arteriovenous Malformation; mRS, modified Rankin Scale*.

* *Statistical significance (P <0.05)*.

### Factors Associated With Long-Term Disability

**Figure 2:**
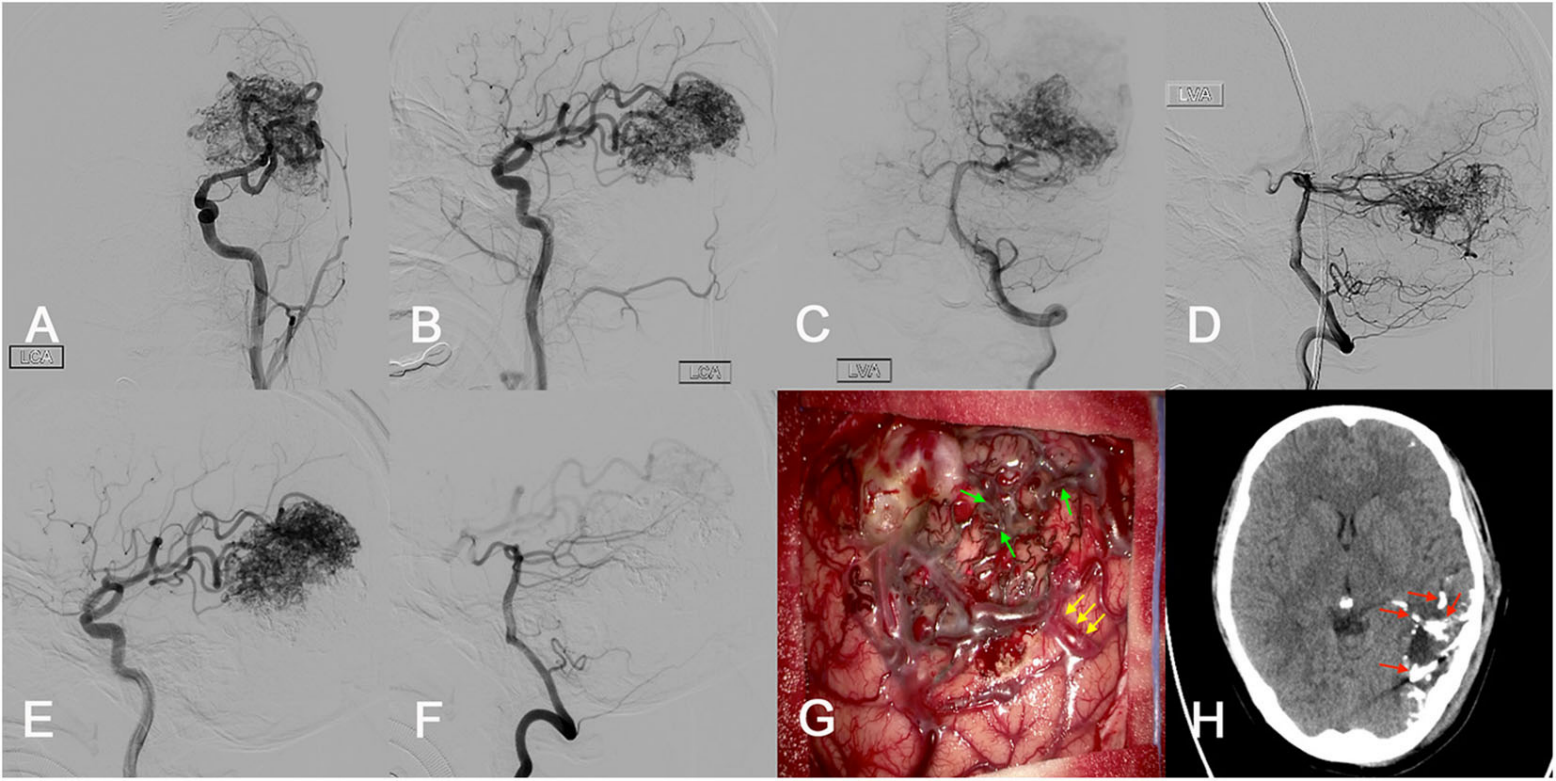
Illustration case. A 25-year-old female presented with loss of consciousness accompanied by physical convulsions 10 days before admission. **(A–D)** The preoperative imaging examination suggested an unruptured AVM in the left parietal occipital lobe (SM grade IV). The maximum diameter was 7.1 cm. The nidus was supplied by the ipsilateral middle cerebral artery (MCA) and posterior cerebral artery (PCA). The nidus was compact. She received a single-stage combined embolization and resection 4 days after admission. **(E,F)** The intraoperative embolization was mainly performed on the nidus supplied by the ipsilateral PCA, and the overall embolization degree was Grade B (30–60%). **(G)** After craniotomy, the main feeding artery was controlled and blocked with aneurysm clips (main feeding artery: yellow arrow). The embolized vessels and lesions appear black (embolized lesions: green arrow), which make the lesion boundary easy to be identified. **(H)** Intraoperative DSA and postoperative CT showed completely obliteration of the lesion. In order to preserve important neurological functions, a small number of embolized lesions adjacent to the eloquent area were maintained during resection (maintained embolized lesion: red arrow). Immediate DSA after the microsurgical resection procedure demonstrated complete obliteration of the lesion. The intraoperative blood loss was 200 ml and the resection duration was 2.9 h. After 2.4 years' follow-up, she experienced no neurofunctional deficit and mRS score of 0.

Univariate analysis revealed that diffuse nidus (*P* = 0.002) and deep perforating arteries (*P* = 0.012) contributed to long-term disability (mRS > 2) of the 5 patients (10.6%) in the hybrid group. Excessive embolization (Grade C: embolize 60–100%) may be associated with long-term disability (*P* = 0.073). However, multivariable logistic regression analysis revealed that excessive embolization (*P* = 0.041; odds ratio, 24.000; 95% CI, 1.140–505.194) was significantly associated with long-term disability in the hybrid group (Table 3). Further subgroup analysis showed that the embolization degree significantly correlated with intraoperative blood loss (*P* = 0.033). Compared with minimal embolization (Grade A, 0–30%), moderate embolization (Grade B, 30–60%) could significantly decreases intraoperative blood loss (*P* = 0.009; odds ratio, 157.64; 95% CI, 110.068–745.487; Bonferroni correction: *P* = 0.018) (Figure 2). No significant differences were found in short-term (*P* = 0.213) and long-term mRS (*P* = 0.228) between different embolization degrees (Table 4). However, there was a trend of higher disability rate in the excessive embolization group (Grade C; 20.0 vs. 0–5.6%, *P* = 0.128).

**Table 3:** Risk factor analysis of long-term disability (mRS > 2) in the whole AVM cohorts.

**Characteristics**	**Univariable**		***P*-value**	**Multivariable**		***P*-value**
	**Present**	**Absent**		**OR**	**95% CI**	
Hybrid group	*n* = 5	*n* = 42				
Sex (male)	2 (40.0%)	24 (57.1%)	0.466			
Age (years)	32.0 ± 12.5	29.4 ± 11.7	0.645			
Hypertension	0 (0.0%)	2 (4.8%)	0.618			
Onset manifestation			0.973			
Hemorrhage	3 (60.0%)	23 (54.8%)	0.824			
Seizure	1 (20.0%)	10 (23.8%)	0.849			
Others	1 (20.0%)	9 (21.4%)	0.941			
Admission mRS	1.2 ± 0.4	1.2 ± 0.4	0.942			
Side (left)	2 (40.0%)	23 (54.8%)	0.532			
Size (cm)	5.1 ± 2.1	4.6 ± 1.2	0.433			
Eloquence	4 (80.0%)	31 (73.8%)	0.764			
Deep venous drainage	3 (60.0%)	24 (57.1%)	0.903			
Aneurysms (flow-related)	2 (40.0%)	16 (38.1%)	0.934			
Diffuse nidus	5 (100.0%)	12 (28.6%)	0.002^*^	4.5 × 10^8^	0.000–	0.997
Deep perforating arteries	5 (100.0%)	17 (40.5%)	0.012^*^	4.8 × 10^8^	0.000–	0.997
Embolization degree			0.128			
Grade A	0 (0.0%)	9 (21.4%)	0.250			
Grade B	1 (20.0%)	17 (40.5%)	0.373			
Grade C	4 (80.0%)	16 (38.1%)	0.073	24.000	1.140–505.194	0.041^*^
Surgical resection alone group	*n* = 7	*n* = 46				
Sex (male)	5 (71.4%)	28 (60.9%)	0.591			
Age (years)	32.1 ± 11.9	31.6 ± 15.4	0.934			
Hypertension	0 (0.0%)	4 (8.7%)	0.965			
Onset manifestation			0.377			
Hemorrhage	5 (71.4%)	22 (47.8%)	0.245			
Seizure	2 (28.6%)	16 (34.8%)	0.746			
Others	0 (0.0%)	8 (17.4%)	0.231			
Admission mRS	1.3 ± 0.5	1.2 ± 0.5	0.681			
Side (left)	3 (42.9%)	25 (54.3%)	0.570			
Size (cm)	3.8 ± 1.5	5.0 ± 1.6	0.064	0.948	0.888–1.013	0.112
Eloquence	6 (85.7%)	39 (84.8%)	0.949			
Deep venous drainage	5 (71.4%)	15 (32.6%)	0.048^*^	1.461	0.170–12.583	0.730
Aneurysms (flow-related)	2 (28.6%)	9 (19.6%)	0.584			
Diffuse nidus	3 (42.9%)	9 (19.6%)	0.170			
Deep perforating arteries	6 (85.7%)	13 (28.3%)	0.003^*^	15.389	1.412–167.669	0.025^*^

*AVM, Arteriovenous Malformation; CI, Confidence Interval; mRS, modified Rankin Scale; OR, odds ratio*.

* *Statistical significance (P <0.05)*.

**Table 4:** Comparison of clinical outcomes between different embolization degrees in the hybrid group.

**Characteristics**	**Embolization degree**			***P* value**
	**Grade A (0–30%) (*n* = 9)**	**Grade B (30–60%) (*n* = 18)**	**Grade C (60–100%) (*n* = 20)**	
Sex (male)	5 (55.6%)	8 (44.4%)	13 (65.0%)	0.443
Age (years)	25.3 ± 11.8	27.5 ± 9.8	33.7 ± 12.4	0.119
Hypertension	0 (0.0%)	0 (0.0%)	2 (10.0%)	0.170
Onset manifestation				0.086
Hemorrhage	7 (77.8%)	7 (38.9%)	12 (60.0%)	0.128
Seizure	2 (22.2%)	4 (22.2%)	5 (25.0%)	0.976
Others	0 (0.0%)	7 (38.9%)	3 (15.0%)	0.021^*^
Admission mRS	1.3 ± 0.5	1.1 ± 0.3	1.3 ± 0.4	0.374
Side (left)	6 (66.7%)	8 (44.4%)	11 (55.0%)	0.535
Size (cm)	5.2 ± 1.5	4.9 ± 1.3	4.2 ± 1.2	0.132
Eloquence	8 (88.9%)	15 (83.3%)	12 (60.0%)	0.137
Deep venous drainage	4 (44.4%)	10 (55.6%)	13 (65.0%)	0.572
Spetzler-Martin Grade				0.058
III	3 (33.3%)	8 (44.4%)	15 (75.0%)	
IV	6 (66.7%)	8 (44.4%)	5 (25.0%)	
V	0 (0.0%)	2 (11.1%)	0 (0.0%)	
Aneurysms (flow-related)	4 (44.4%)	8 (44.4%)	6 (30.0%)	0.598
Diffuse nidus	5 (55.6%)	4 (22.2%)	8 (40.0%)	0.206
Deep perforating arteries	5 (55.6%)	9 (50.0%)	8 (40.0%)	0.696
Follow-up time (years)	2.3 ± 0.5	2.3 ± 0.7	2.5 ± 0.6	0.604
Operation duration (hours)	7.4 ± 1.9	6.7 ± 2.8	6.5 ± 1.7	0.620
Resection duration (hours)	3.8 ± 1.6	3.2 ± 0.9	3.9 ± 1.3	0.141
Intraoperative blood loss (ml)	916.7 ± 482.2	488.9 ± 347.5	640.0 ± 373.3	0.033^*^
Postoperative intracranial infection	4 (44.4%)	5 (27.8%)	3 (15.0%)	0.240
Postoperative infarction	0 (0.0%)	1 (5.6%)	0 (0.0%)	0.376
Postoperative hemorrhage	0 (0.0%)	1 (5.6%)	2 (10.0%)	0.452
Perioperative subsequent surgery	0 (0.0%)	1 (5.6%)	0 (0.0%)	0.376
New-onset visual disturbance	0 (0.0%)	1 (5.6%)	1 (5.0%)	0.645
New-onset aphasia	1 (11.1%)	1 (5.6%)	0 (0.0%)	0.281
New-onset Weakness	1 (11.1%)	2 (11.1%)	4 (20.0%)	0.702
Short-term mRS	1.4 ± 0.7	1.5 ± 0.9	2.0 ± 1.2	0.213
Long-term obliteration rate	100.0%	100.0%	100.0%	1.000
Long-term mRS	0.4 ± 0.7	0.5 ± 0.9	1.0 ± 1.2	0.228
Long-term disability (mRS > 2)	0 (0.0%)	1 (5.6%)	4 (20.0%)	0.128
Death	0 (0.0%)	0 (0.0%)	0 (0.0%)	1.000

*mRS, modified Rankin Scale*.

* *Statistical significance (P <0.05)*.

In the surgical resection alone group, 7 patients (13.2%) suffered long-term disability (mRS > 2). Deep venous drainage (*P* = 0.048) and deep perforating arteries (*P* = 0.003) were significantly associated with long-term disability in the univariate analysis. Multivariable logistic regression analysis revealed that deep perforating arteries (*P* = 0.025; odds ratio, 15.389; 95% CI, 1.412–167.669) were significantly associated with long-term disability (Table 3).

## Discussion

The ideal treatment strategy for SM grade III–V AVMs remains controversial (4, 5), but the single-stage combined embolization and microsurgical resection strategy might be potentially effective for complex AVMs. In this study, we reviewed 100 consecutive AVM patients (47 corrected using hybrid procedures whereas 53 by surgical resection alone) who were followed up for an average of 2.3 years, and we found no significant differences between these two interventional strategies with regard to long-term outcomes (both in the ruptured and unruptured cohorts). However, the hybrid strategy significantly accelerated the resection duration. Excessive embolization (Grade C, 60–100%) was an independent risk factor associated with long-term disability (mRS) in the hybrid group as opposed to involvement of deep perforating artery in the surgical resection alone group. Subgroup analysis of hybrid cohorts further revealed that moderate embolization (Grade B, 30–60%) resulted in better outcome.

Hybrid angio-surgical suite combines the advantages of both embolization and surgical resection, and its effectiveness for the complex cerebrovascular disease has been confirmed by several studies (Table 5) (6–10, 12, 13, 15). Several surgical dynamics could influence the outcome, including (1) intraoperative embolization can reduce the blood flow in the nidus before resection (8, 9), (2) intraoperative embolization can reduce the grade of lesions, (3) intraoperative embolization can eliminate high-risk characteristics and challenging surgical areas, such as aneurysms, fistulas, and deep perforating arteries (16), (4) single-stage strategy can avoid corresponding complications between the staged treatment interval and reduces the risks associated with multiple anesthesia (8, 9), (5) the intraoperative angiogram after resection enables neurosurgeons to quickly confirm the complete obliteration (16), (6) single-stage strategy could reduce multiple treatment cost (9), (7) hybrid surgery offers unique advantages in the management of vascular emergency interventions (17). Moreover, our team has indicated that targeted *in situ* embolization could increase the lesion-to-eloquence distance (LED), which could prevent damages to eloquences during surgical resection in our previous study (15). In this research, shorter resection duration demonstrated the superiority of hybrid surgery for AVMs compared with surgical resection alone, because it minimized the surgical related challenges. Intraoperative embolization also can reduce the lesion grade (such as reduction of lesion volume, occlusion of deep drainage, or embolization of brain tissue adjacent to eloquent areas, etc.). These advantages are very important for neurosurgeons, particularly young neurosurgeons with insufficient experience in the operation of high-level AVMs or emergency ruptured AVMs. Besides, intraoperative angiography revealed residual lesion in 1 patient in the hybrid group, and the residual lesion was successfully corrected by a subsequent resection before the skull closure of the first resection, which promoted the superiority of hybrid surgery in preventing residual lesions. Postoperative hemorrhage occurred in three patients (6.4%) during the perioperative period. The incidence was lower than previous reports on hybrid strategy (14.3–22.7%) but consistent with the other single interventional strategies (4.0–12.2%) (8, 9, 18). The underlying mechanism might be the normal perfusion pressure breakthrough (NPPB), since no clear recanalization bleeding was found during the subsequent hematoma removal. As reported elsewhere, five patients (10.6%) experienced postoperative long-term disability (3.2–12.5%) (9, 13).

**Table 5:** Summary of studies on single-stage combined embolization and resection for AVMs.

**Study**	**Number of patients**	**Age (years)**	**Number of ruptured AVM**	**SM grade IV–V**	**Embolization strategy**	**Intraoperative residue detected**	**Obliteration rate (%)**	**Postoperative hemorrhage**	**Recommend hybrid surgery**
(6)	1	NA	NA	NA	NA	NA	100	0/1	Yes
(10)	4	NA	3/4	NA	Nidus embolization	1/4	100	0/4	Yes
(7)	5	NA	NA	NA	NA	NA	100	NA	Yes
(12)	6	NA	6/6	NA	Nidus and feeding artery embolization	0/6	100	0/6	Yes
(13)	8	37.4 (average)	5/8	6/8	Nidus and feeding artery embolization	0/8	100	0/8	Yes
(11)	1	40	1/1	0/1	Retrograde venous embolization	0/1	100	0/1	Yes
(8)	7	NA	NA	NA	Nidus embolization	1/7	100	1/7	Yes
(9)	22	NA	13/22	5/22	Nidus and feeding artery embolization	1/22	95.5	5/22	Yes
(14)	9	30.4 ± 14.8	3/9	3/9	Nidus and feeding artery embolization	0/9	100	0/9	Yes
Present study, 2019	47	29.7 ± 11.7	26/47	21/47	Nidus and feeding artery embolization	1/47	100	3/47	Yes

### Intraoperative Embolization Strategy

Currently, intraoperative cerebrovascular angiography has two applications in hybrid surgery: (1) intraoperative diagnostics and (2) intraoperative embolization. In general, the intraoperative diagnostic angiography was considered to be effective in improving the obliteration rate of lesions and offering fast quality control (19). However, studies on intraoperative embolization strategies are limited (Table 4). At present, there are three main types of intraoperative embolization procedures: (1) nidus embolization, (2) nidus and feeding artery embolization, and (3) retrograde venous embolization. Many previous studies reported that the nidus+feeding artery embolization could provide a bloodless surgical plane for surgical resection, and the embolic agents could be used to mark arterial feeders during the resection procedure (9, 12, 15). Besides, intraoperative retrograde venous embolization was an emerging hybrid strategy for treatment of cerebral AVMs and has been postulated to achieve a high rate of complete AVM obliteration and excellent functional outcomes (11, 14). In this study, we adopted the nidus+feeding artery embolization strategy. After embolization with Onyx, the embolized lesions appear black, and the feeding arteries and lesion boundary could easily be identified in the subsequent resection procedure.

To our knowledge, the relationship between the embolization degree and clinical prognosis for the hybrid surgery has not been fully evaluated. One previous study suggested that simultaneous occlusion of more than 60% of AVM volume could induce significant redistribution of local blood flow (20), which might develop secondary NPPB and hyperemia of the adjacent brain (21). Besides, the increased pressure of feeding artery after embolization was reported to be proportional to the degree of AVM obliteration (22). Excessive blood stasis might occur in the residual lesions when the increased feeding artery pressure transmitted to the residual nidus, which might increase the intranidal pressure and finally lead to the subsequent rupture. Therefore, limited embolization was recommended to avoid excessive high pressure in the feeding arteries (23). In this study, we found excessive embolization (Grade C, 60–100%) was significantly associated with the postoperative long-term disabilities in the SM grade III–V AVMs. The underlying mechanism might be the unpredictable acute neurological impairment caused by the intraoperative rupture during the embolization, or difficulty in resecting deep lying lesions caused by the constraint of lifting the rigid embolized nidus, or the intraoperative hemostasis difficulty caused by congestion in the remaining lesions after embolization, which damages more functional brain tissues. In addition, the moderate embolization (Grade B, 30–60%) eases subsequent resection by effectively reducing intraoperative blood loss.

### Intraoperative Resection Strategy

Surgical resection is the most critical step in hybrid surgery, and it is controversial whether the embolized lesions should be removed. Kocer et al. (9) suggested that if deemed safe, then the embolized component of the lesion should be completely removed. However, the excision of embolic lesions near the eloquence presents a high risk of neurological impairment. Accordingly, we suggest retention of a properly embolized lesions to maintain a sufficient lesion-to-eloquence distance as recommended in a previous study (15). Even so, there is a 0 to 13% risk of recanalization in the embolized occluded lesion after the operation (24–26). In this study, we retained the embolized occluded lesions with critical functions monitored by the intraoperative electroencephalogram and somatosensory evoked potential, and the embolized artery residues were repeatedly coagulated to avoid recurrence of AVM. And as expected, no recurrence was observed at the last follow-up.

Our study design presented potential limitations. Firstly, because this is a single-center retrospective study, the operative indicators and intraoperative manipulation may vary across institutions. In addition, although the degree of intraoperative embolization in this study was evaluated by three experienced neuroradiologists based on the changes in lesion volume and blood flow, the results were still relatively subjective and lacked quantitative measures. Subsequently, we recommend for further multicenter prospective trials on hybrid strategy to explore the optimal intervention approach for AVMs.

## Conclusions

Single-stage combined embolization and microsurgical resection can provide an efficient strategy for the treatment of SM grade III–V AVMs. However, there are no statistical differences between hybrid strategy and surgical resection alone with regard to short-term and long-term prognosis. However, the hybrid strategy presents significant advantages by reducing the resection duration. Excessive embolization (Grade C, 60–100%) was an independent risk factor for long-term disability in the hybrid group, as opposed to involvement of deep perforating arteries for long-term disability in the surgical resection alone group. In addition, moderate embolization (Grade B, 30–60%) was recommended because of the lower intraoperative blood loss.

## Data Availability Statement

All datasets presented in this study are included in the article/ supplementary material.

## Ethics Statement

The studies involving human participants were reviewed and approved by the ethics committee of Peking University International Hospital. Written informed consent to participate in this study was provided by the participants, and where necessary, the participants' legal guardian/next of kin.

## Author Contributions

YC conceived the idea, designed the paper, and wrote the manuscript. RL and LM performed the statistical analysis. RL, YZ, and TY collected the data and designed the paper. XY, XC, HW, RW, and YLZ funded the study, critically revised the manuscript and approved the final manuscript as submitted. All authors agreed to be accountable for all aspects of the work in ensuring that questions related to the accuracy or integrity of any part of the work are appropriately investigated and resolved.

## Conflict of Interest

The authors declare that the research was conducted in the absence of any commercial or financial relationships that could be construed as a potential conflict of interest.
